# Breast Fistula Repair after Autologous Fat Graft: A Case Report

**DOI:** 10.1155/2011/547387

**Published:** 2011-06-07

**Authors:** Francesco Maria Klinger, Fabio Caviggioli, Davide Forcellini, Valeriano Vinci, Luca Maione, Giorgio Pajardi, Marco Klinger

**Affiliations:** ^1^Università degli Studi di Milano and MultiMedica Holding S.p.A., U.O.C Chirurgia Plastica 2, Via Milanese, 300—20099 Sesto San Giovanni, Milano, Italy; ^2^Università degli Studi di Milano and IRCCS Istituto Clinico Humanitas, 20089 Rozzano, Milano, Italy

## Abstract

We report the case of a 55-year-old female patient who attended our clinic for the presence of a scar retraction in the upper pole of the left breast as a complication of breast augmentation. In the scar area, we observed an orifice that probing revealed to be a fistula. The patient was referred to surgical intervention under general anesthesia to obtain scar contracture release using autologous fat graft; one month after autologous fat injection, following healing of the fistula, the patient underwent a second surgical procedure to replace the left breast implant. Unexpectedly, two weeks after the surgical procedure, complete healing of the breast fistula within the scar area was observed; this observation was confirmed during the second surgical step for left breast implant repositioning, when we observed the absence of the fistula orifice in the breast implant cavity. Upon clinical examination at 1-year followup, tissue integrity was preserved. The patient's satisfaction was excellent. We have observed a possible additional effect of fat graft.

## 1. Introduction

To our knowledge, a significant increase in the number of interventions for breast augmentation has occurred in recent years. Unfortunately, however, complications related to this procedure, including capsular contracture and implant malposition, have not decreased; less frequent, but more serious complications, are infection and extrusion of breast implant [1].

## 2. Case Presentation

We present the case of 55-year-old woman who attended our clinic for a scar retraction in the upper pole of the left breast; the scar retraction occurred as a complication of a breast augmentation previously performed elsewhere. The patient also reported that the left breast implant had been removed about 6 months before by the same surgeon who performed the breast augmentation to allow the healing of the fistula.

**Figure 1:**
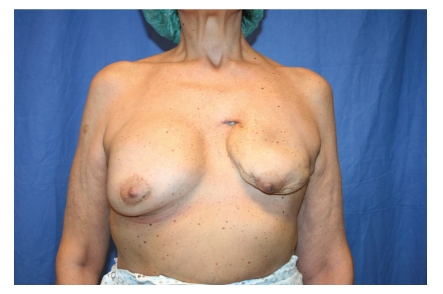
Breast fistula before fat graft.

**Figure 2:**
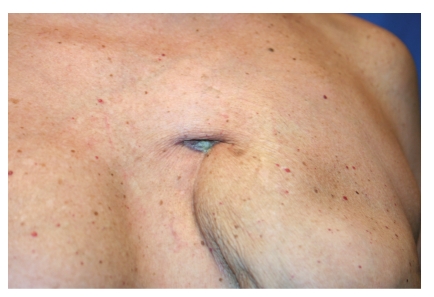
Breast fistula before fat graft (detail).

During breast clinical examination, we observed the presence of an orifice within the scar retraction area. Probing revealed the presence of a fistula (about 4 cm in length) (Figures 1 and 2), which the patient reported being aware of for six months. For the identification of the exact location and length of the breast fistula, fistulography was performed; this examination showed a 4-cm-long tract extending from the skin of the left breast scar area to the retropectoral plane. 

No clinical signs of infection were present, but we decided for antibiotic prophylaxis (amoxicillin + clavulanic acid 1 g bid). After routine preoperative examinations, the patient was referred to a surgical intervention under local anaesthesia to obtain scar contracture release using autologous fat graft, in accordance with our observations [2–7]. Liposuction of the subumbilical area was performed after tumescent infiltration of 100 mL saline solution, 75 mg of levobupivacaine, 40 mg of mepivacaine, and 0.5 mL epinephrine 1 : 1000. Adipose tissue sample of about 10 mL was obtained and processed following Coleman's technique (i.e., centrifuged at 3000 rpm for 5 minutes). Adipocyte cell fraction was isolated.

At the left breast level, in the scar area, a volume of about 2.5 mL was equally injected into the dermal-subdermal junction using a 18-gauge angiographic needle with a snap-on wing (by Cordis, a Johnson & Johnson Company, N.V, Roden, Netherlands). The scar area was not removed but only infiltrated with autologous fat graft, according to observations in scar remodeling [2–6] to avoid distortion of the breast shape; the aesthetic quality improvement of scar area is guaranteed by the effects of fat graft. Following surgery antibiotic therapy was administered for 5 days (cefixime 400 mg tablets). 

One months after healing of the fistula, the patients underwent a second surgical procedure, to replace the left breast implant.

**Figure 3:**
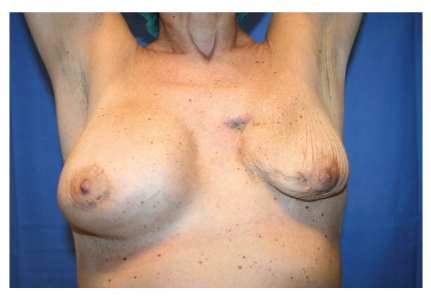
Breast fistula 20 days after fat graft.

**Figure 4:**
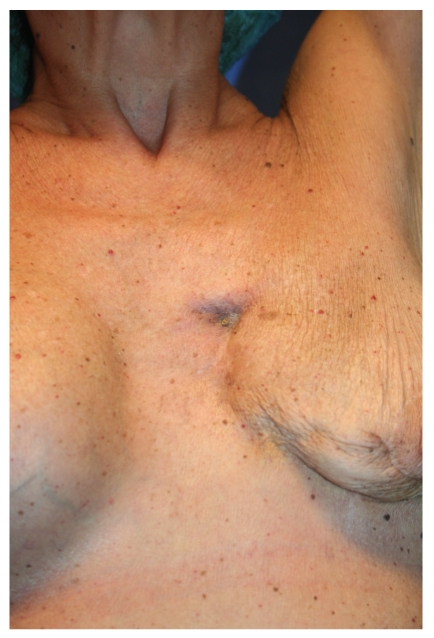
Breast fistula 20 days after fat graft (detail).

Clinical assessment was performed one and two weeks after autologous fat injections. During the second clinical assessment other than the partial release of the scar, unexpected complete healing of the breast fistula within the scar area was observed (Figures 3 and 4); no local or systemic signs of infection were found; no complications occurred.

One month after autologous fat injection, we proceeded with capsulotomy and replacement of the left breast implant (325 g), as in the controlateral side. In course of surgery, we observed the complete absence of the fistula orifice in breast implant cavity at the site described by previous fistulography, confirming the complete healing of the breast fistula.

At 1-year followup clinical examination, tissue integrity was preserved. Patient's satisfaction was excellent.

## 3. Discussion

The rapid improvement in breast fistula healing within the scar areas suggests that there are biological interactions between transplanted fat and local structures, in keeping with our results obtained in reepithelization of posttraumatic ulcers [3]. Fat grafting improves the healing of hard-to-heal wounds, leading to results that would be difficult to achieve with other surgical techniques. 

However, remains to be demonstrated with more detailed studies, what is the role of fat graft in the wound healing process and what are the biological interactions underlying this phenomenon. In this case report, a nonhealing breast fistula, which was present for more than 6 months, is completely healed in only two weeks using fat grafting. We also found the over-time stability of the result.

In accordance with our experience in autologous fat graft [2–6], we observed a possible additional effect of this surgical procedure; indeed, our starting point was the treatment of the scar area with autologous fat graft, in order to obtain relaxation of scarring and allow the repositioning of the breast implant; in addition, we obtained complete healing of breast fistula, which represents a serious problem for breast reimplantation.

## Figures and Tables

**Figure 5:**
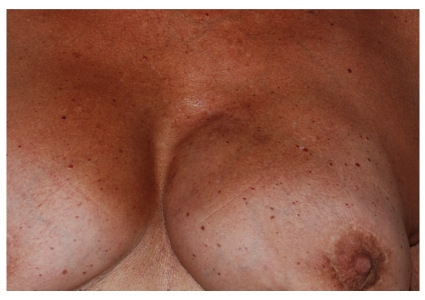
Breast fistula 1-year after fat graft (detail).
